# Draft Genome Sequence of *Rhizobium* sp. Strain T2.30D-1.1, Isolated from 538.5 Meters Deep on the Subsurface of the Iberian Pyrite Belt

**DOI:** 10.1128/MRA.01098-18

**Published:** 2018-10-25

**Authors:** R. García, José M. Martínez, T. Leandro, R. Amils

**Affiliations:** a Centro de Biología Molecular Severo Ochoa (CBMSO, CSIC-UAM), Universidad Autónoma de Madrid, Cantoblanco, Madrid, Spain; b Centre for Neuroscience and Cell Biology, University of Coimbra, Coimbra, Portugal; c Centro de Astrobiología (CAB, CSIC-INTA), Torrejón de Ardoz, Madrid, Spain; University of Arizona

## Abstract

*Rhizobium* sp. strain T2.30D-1.1 was isolated from the deep subsurface of the Iberian Pyrite Belt. We report its draft genome, consisting of 60 contigs with a chromosome of ≈4.6 Mb and a plasmid of 179 kb. The annotation revealed 4,526 coding DNA sequences, 45 tRNA genes, and 1 rRNA operon.

## Abstract (precis)

*Rhizobium* sp. strain T2.30D-1.1 was isolated from the deep subsurface of the Iberian Pyrite Belt.

## ANNOUNCEMENT

The genus *Rhizobium* is classified within the family *Rhizobiaceae*, class *Alphaproteobacteria*. *Rhizobium* species have been isolated from soil environments, and most are able to establish symbiosis with leguminous plants and are characterized as Gram-negative aerobic bacteria (1). The facultative *Rhizobium* sp. strain T2.30D-1.1 was isolated from a strict anaerobic denitrification enrichment culture using a 538.5-m-deep core sample obtained from a devoted geomicrobiological drilling project (ERC 250350-IPBSL) aimed at studying the microbially diverse subsurface populations of the Iberian Pyrite Belt in southwest Spain (2). The Iberian Pyrite Belt is one of the largest known sulfidic ore deposits in the world (3). Drilling was performed as described by Puente-Sánchez et al. (4). The coordinates of the drilling borehole were 37^°^43ʹ45.42ʺN, 6^°^33ʹ23.57ʺW. A powdered rock sample (≈6 g) was used as an inoculum. The anaerobic culture medium for enrichment of chemolithoautotrophic denitrifying microorganisms consisted of a basal medium (per liter of distilled water, 0.3 g NH_4_Cl, 0.3 g K_2_HPO_4_·3H_2_0, 0.1 g MgSO_4_·7H_2_0, 0.4 g NaHCO_3_, 0.01 g CaCL_2_·2H_2_0, 0.1 g yeast extract, and 1 ml trace element solution [5] at an initial pH of 7) to which 40 mM nitrate was added as an electron acceptor and 20 mM thiosulfate with H_2_-CO_2_ (80:20, vol/vol) was added as a possible electron donor. The enrichment was incubated at 30°C in the dark without shaking for 1 year. Activity was measured by the disappearance of nitrate at different intervals. Isolation of anaerobic microorganisms was performed using the Hungate roll tube method (6). The culture medium for isolation of anaerobes had the same composition as the enrichment culture medium, with the addition of Noble agar (15 g/liter) (Difco Laboratories) as a solidifying agent. Hungate roll tubes were inoculated with serial dilutions of the enrichment culture. Incubation was at 30°C in the dark until development of isolated colonies. Colonies were picked in anaerobic conditions with a bent Pasteur pipette and transferred onto culture plates with the same cultivation conditions. Plates were incubated in anaerobic jars with AnaeroGen sachets (Oxoid) to generate anaerobic conditions, and the gas mixture (H_2_-CO_2_, vol/vol) was injected inside the jar. Subculturing was done for at least three transfers. Colonies were screened for unique morphologies, and representatives of each one were selected for further identification.

Genomic DNA of strain T2.30D-1.1 was extracted from a culture in R2A liquid medium (7) using a phenol-chloroform protocol (8). DNA was amplified using the universal 16S rRNA gene primers 27F (5ʹ-AGAGTTTGATCATGGCTCAG-3ʹ) and 1492R (5′-GGTTACCTTGTTACGACTT-3′). PCR products were checked on 1% agarose gel purified with a JetQuick DNA purification kit (GenoMed) and sequenced by the Unidad de Secuenciación y Bioinformática of Centro de Astrobiología (INTA–CSIC, Spain) with the same primers. Reads were edited and assembled using MEGA7 and BioEdit. Finally, the complete 16S rRNA gene sequences were compared to those in the GenBank database of the National Center for Biotechnology Information with BLAST. The closest sequence was found to be Rhizobium selenitireducens (98.96%), a strictly aerobic bacterium (9).

The concentration of genomic DNA was determined with a Qubit version 2.0 fluorometer (Invitrogen, USA), and its quality was checked with electrophoresis in 1% agarose gel (wt/vol) (Conda, Spain) in 0.5× Tris-borate-EDTA (TBE) buffer. The MicrobesNG sequencing service at the University of Birmingham sequenced the genomic DNA using the Illumina MiSeq platform with 30× coverage. The sequencing run generated 736,223 paired-end reads with a mean length of 250 bp forward and 210 bp reverse. *De novo* assembly was carried out using the software SPAdes version 3.9.0 (default parameters with the “–careful” option) (10), and assembly of extrachromosomal genetic elements was performed using plasmidSPAdes (default parameters with the “–careful” option), which uses the read coverage of contigs to distinguish between plasmids and chromosomes (11). These plasmid contigs were aligned against the chromosomal assembly with the Mauve aligner (12) in order to separate chromosomal and plasmid contigs. Contigs were ordered and new scaffolds were created by means of SSPACE software (default parameters) (13). This yielded a chromosome in 60 scaffolds with an *N*_50_ value of 361 kb, a GC content of 63.13%, and 4,658,326 bp and a plasmid of 179 kb in a single contig. Comparable plasmid sizes have been reported for other members of the genus *Rhizobium* (150 to 400 kb) (14).

Gene prediction and annotation were carried out using the RAST platform (15) taking Rhizobium leguminosarum bv. viciae 3841 as a reference genome. A total of 4,526 coding DNA sequences, 45 tRNA genes, and 1 rRNA operon were identified. In addition, a complete set of genes encoding (i) sulfur oxidation (SoX), nitrate ammonification, and denitrification, (ii) a series of highly conserved reductase enzymes (DSRs) which are part of terminal electron acceptors in anaerobic respiration, (iii) resistance to heavy metals (Co, Zn, Cd, Cu and As), (iv) flagellar motility, and (v) chemotaxis were identified in the chromosome.

Regarding annotation of the plasmid, 195 coding DNA sequences were identified. Genes related to the interaction with plants were detected, specifically those related to virulence (*vir* genes), in addition to genes involved in plasmid replication and the conjugative transfer process.

The comprehensive analysis of the genome of *Rhizobium* sp. strain T2.30D-1.1 should provide insights on the mechanisms used to inhabit deep terrestrial anaerobic environments under oligotrophic conditions and in the absence of light.

### Data availability.

Reads have been deposited in DDBJ/ENA/GenBank under the SRA accession number ERR2572852, and the complete genome sequences and annotations have been deposited under the accession numbers UEYP00000000 (for this chromosome) and LS974446 (for this plasmid). This version described in this paper is the first version.
